# An embedded system for the automated generation of labeled plant images to enable machine learning applications in agriculture

**DOI:** 10.1371/journal.pone.0243923

**Published:** 2020-12-17

**Authors:** Michael A. Beck, Chen-Yi Liu, Christopher P. Bidinosti, Christopher J. Henry, Cara M. Godee, Manisha Ajmani

**Affiliations:** 1 Department of Physics, University of Winnipeg, Winnipeg, Manitoba, Canada; 2 Department of Applied Computer Science, University of Winnipeg, Winnipeg, Manitoba, Canada; 3 Department of Electrical and Computer Engineering, University of Manitoba, Winnipeg, Manitoba, Canada; 4 Department of Biology, University of Winnipeg, Winnipeg, Manitoba, Canada; Korea National University of Transportation, REPUBLIC OF KOREA

## Abstract

A lack of sufficient training data, both in terms of variety and quantity, is often the bottleneck in the development of machine learning (ML) applications in any domain. For agricultural applications, ML-based models designed to perform tasks such as autonomous plant classification will typically be coupled to just one or perhaps a few plant species. As a consequence, each crop-specific task is very likely to require its own specialized training data, and the question of how to serve this need for data now often overshadows the more routine exercise of actually training such models. To tackle this problem, we have developed an embedded robotic system to automatically generate and label large datasets of plant images for ML applications in agriculture. The system can image plants from virtually any angle, thereby ensuring a wide variety of data; and with an imaging rate of up to one image per second, it can produce lableled datasets on the scale of thousands to tens of thousands of images per day. As such, this system offers an important alternative to time- and cost-intensive methods of manual generation and labeling. Furthermore, the use of a uniform background made of blue keying fabric enables additional image processing techniques such as background replacement and image segementation. It also helps in the training process, essentially forcing the model to focus on the plant features and eliminating random correlations. To demonstrate the capabilities of our system, we generated a dataset of over 34,000 labeled images, with which we trained an ML-model to distinguish grasses from non-grasses in test data from a variety of sources. We now plan to generate much larger datasets of Canadian crop plants and weeds that will be made publicly available in the hope of further enabling ML applications in the agriculture sector.

## 1 Introduction

A review of the recent literature shows there is great optimism that advances in sensors [1–4], robotics [5–10], and machine learning [11–16] will bring new innovations destined to increase agricultural production and global food security. Whether one speaks more broadly of precision agriculture, digital agriculture, smart farming, or Agriculture 4.0 (in reference to the anticipated fourth agricultural revolution), the confluence of these technologies could lead, for example, to automated methods of weeding, disease evaluation, plant care, phenotyping, and yield predictions [16–28]. Such capabilities would increase crop yields and expedite breeding programs, while minimizing inputs (e.g. water, fertilizer, herbicide, pesticide) and reducing the impact on the environment.

Prototypes of autonomous vehicles performing farming tasks in the field exist already [9, 10, 29, 30]. However, putting the “brains” into such agents is still a hard challenge and success is limited to a crop’s specifics and the task at hand. Machine learning (ML) utilizing convolutional neural networks (CNNs) holds great promise for image-based location and identification tasks in agriculture. The capabilities of CNNs have improved vastly in recent years [31–33] and are now used as solutions to previously difficult problems such as object detection within images [34], facial recognition [35], automatic image annotation [36], self-driving cars [37] and automated map production [38].

While there are many different CNN architectures and training methods, a general rule of thumb is the following: A model’s capability to identify objects in previously unseen data (called generalizing) depends significantly on the amount of data the model *has seen* during training [31, 39]. As a result, an inadequate amount of high-quality training data—in particular, labeled data—is often the bottleneck in developing ML-based applications, a fact underscored by many authors working in plant sciences and agriculture [11–13, 17–19, 21–26]. This problem is magnified by the circumstance that each application is likely to require its own specific training data, especially given the very wide variety of plant appearances, e.g. tillering versus ripening, healthy versus diseased, crop versus weed. For example, training CNNs to distinguish oats from their wild counterpart—which are responsible for an annual loss of up to $500 million in the Province of Manitoba alone (according to https://www.gov.mb.ca/agriculture/crops/weeds/wild-oats.html)—would certainly require a qualitatively and quantitatively rich dataset of labeled images of all variants.

The need for labeled training data is often satisfied by manual annotation, which is typically achieved through one of two ways. If the classification problem is common knowledge, it can be crowdsourced, as is done through platforms, such as Mechanical Turk [40] and ReCaptcha [41]. Conversely, if the classification problem requires expert knowledge, crowdsourcing will not be reliable and annotation must be performed by experts only. Both methods have been suggested for labeling plant images [12, 22, 24, 25], and although there are tools available to ease the process [42–44], manual annotation is both cost- and time-intensive and usually leads to comparably small datasets in the magnitude of a couple of thousands images. As a workaround to having large, labeled datasets, several strategies, such as transfer learning with smaller labeled data sets [12, 45, 46] or unsupervised learning with unlabeled data [18], are being explored.

Variety in a dataset can also be increased by data augmentation, i.e. modificationss like rotating, translating, or color correction of existent images [12]. Which of these are suitable for a given dataset can be determined manually for each dataset or be incorportated into the learning process itself [47]. While including data augmentation can enrich an existing dataset in the training process and thus increase the robustness of the resulting neural network, it cannot fully resolve the need to generate actual images of plants from different perspectives, since, for example, plants have fundamentally different features when looked at from above or in profile. Another interesting approach is to generate synthetic images via plant models [11, 12, 46], generative adversarial networks [48, 49] or by merging images belonging to the same class (“smart augmentation”) [50]. To apply data augmentation and generative adversarial networks, an initial basis of labeled data is required. Similarly, for generating synthetic images a plant cultivar must be modeled such that the resulting images match the phenological properties of real plants. We cannot use data augmentation or synthetic methods to create, say, images of corn plants from a sunflower dataset. In other words these methods do not create images of classes different to those originally contained in the dataset. Thus, they do not scale over the wide variety of different crops and weeds and their respective growing stages. The ability to generate labeled images directly from real objects, then, remains a matter of great importance for machine learning in general.

In an effort to produce large quantities of high-quality training data for ML applications in agriculture, we have developed an embedded system to automatically generate and label images of real plants. This system—henceforth referred to as EAGL-I (**E**mbedded **A**utomated **G**enerator of **L**abeled **I**mages)—is, in a nutshell, a robotically moved camera that takes pictures of known plants at known locations from a large variety of known positions and angles. This allows us to collect a wider variety of images of a single plant. We can, for example, take top-down images or rotate the camera around the plant taking profile shots from any angle, allowing us to capture its three-dimensional features. This cannot be achieved by simple linear transformations of images captured from a fixed perspective.

Since we have full information and control over where on the image the plants are located, we can automatically identify and label them. As a result, EAGL-I can generate labeled data at the rate of thousands to tens of thousands of images per day, with minimal human interaction and no dependence on crowdsourcing or expert knowledge. This allows us to generate initial datasets, comprising of up to 20,000 images, for a specific crop or weed within a single day. Imaging over several days allows us to add variety to the dataset by adding more individuals of the same cultivar and by tracking their growing stages. With the EAGL-I system we can thus generate labeled plant datasets for machine learning applications on demand and in large quantities. The system described here can be applied to virtually any machine learning application and scaled to meet the needs at hand.

**Fig 1:**
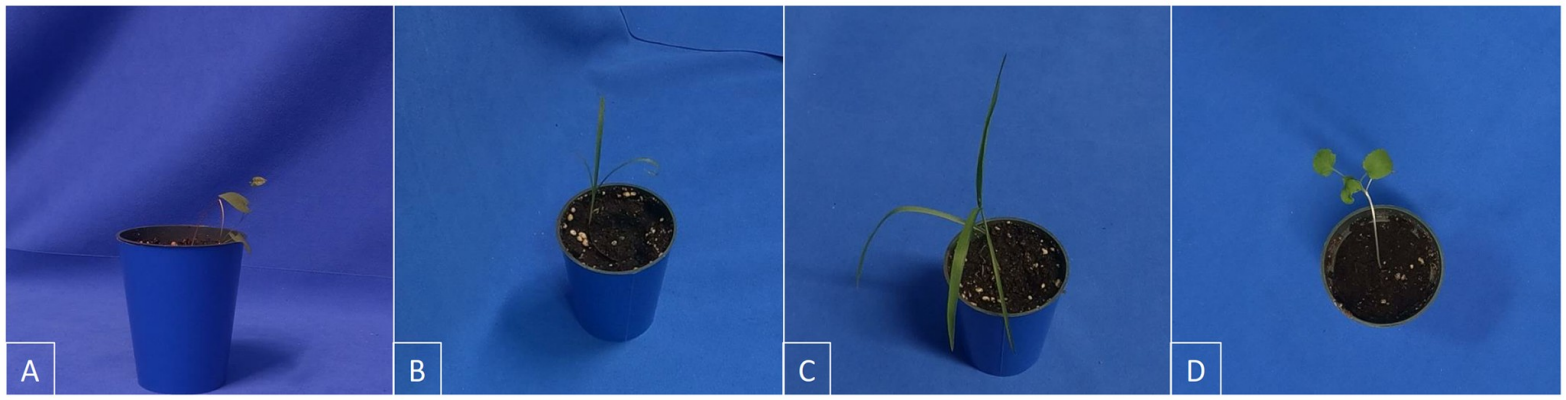
Example images taken by EAGL-I. A: Wild buckwheat in a profile shot. B-C: Yellow foxtail and barnyard grass in oblique angles. D: Canola in an overhead shot. Blue keying fabric is used as background.

While there are many examples in the literature of plant imaging systems already [51–64], their primary purpose has been to capture and compare phenotypic information and growth metrics. This is typically achieved through overhead shots only and requires close-to-zero variance in imaging conditions to ensure a high accuracy in extracting plant characteristics. This is at odds with the type of datasets needed to train machine learning algorithms for plant classification. In this case, one is interested in a *rich* dataset, with a wide variety of images falling under the same label. Variety can be achieved through differences in the chosen parameters, such as imaging angle (Fig 1), camera-to-plant distance, lighting conditions, time of day, growth stage, and the use of different plants of the same cultivar or species. One must also include different plants with different growing characteristics. For example corn (a fast-growing, tall grass) is very different, say, compared to dandelion (a ground-hugging rosette), but one still needs examples of both (and indeed others) in the same training set to identify crop versus weed with the highest possible accuracy. EAGL-I has the capabilities to incorporate all these differences and is, to the best of our knowledge, the only imaging system fully dedicated to the goal of generating machine learning datasets for plant classification.

The contributions of this paper are the following:

- We designed an imaging system to create labeled datasets for training machine learning models
- This system has a high imaging rate and autonomously labels the imaged plants, offering an alternative to time- and cost-intensive manual labeling
- The system can image plants from any angle and at different distances, thus, exceeding simple image augmentation and producing the variety needed for training datasets (see [65, 66])
- A wide variety of plants can be imaged and there is full freedom in their arrangement in the coverable volume
- As a proof of concept, we generated a dataset of different weeds commonly found in the Province of Manitoba, trained a CNN with it, and evaluated the resulting model on previously unseen data

The rest of the paper is structured as follows. Section 1 describes the EAGL-I’s parts, specifications, and mode of operation. Section 2 describes data generation and defines the imaging rate of EAGL-I. It also lists the parameters we used in production to generate a training dataset. In Section 3, we characterize that dataset and use it to train a CNN to distinguish dicots from monocots. Section 4 concludes the paper and discusses planned improvements to the system and future work.

## 2 System overview

**Fig 2:**
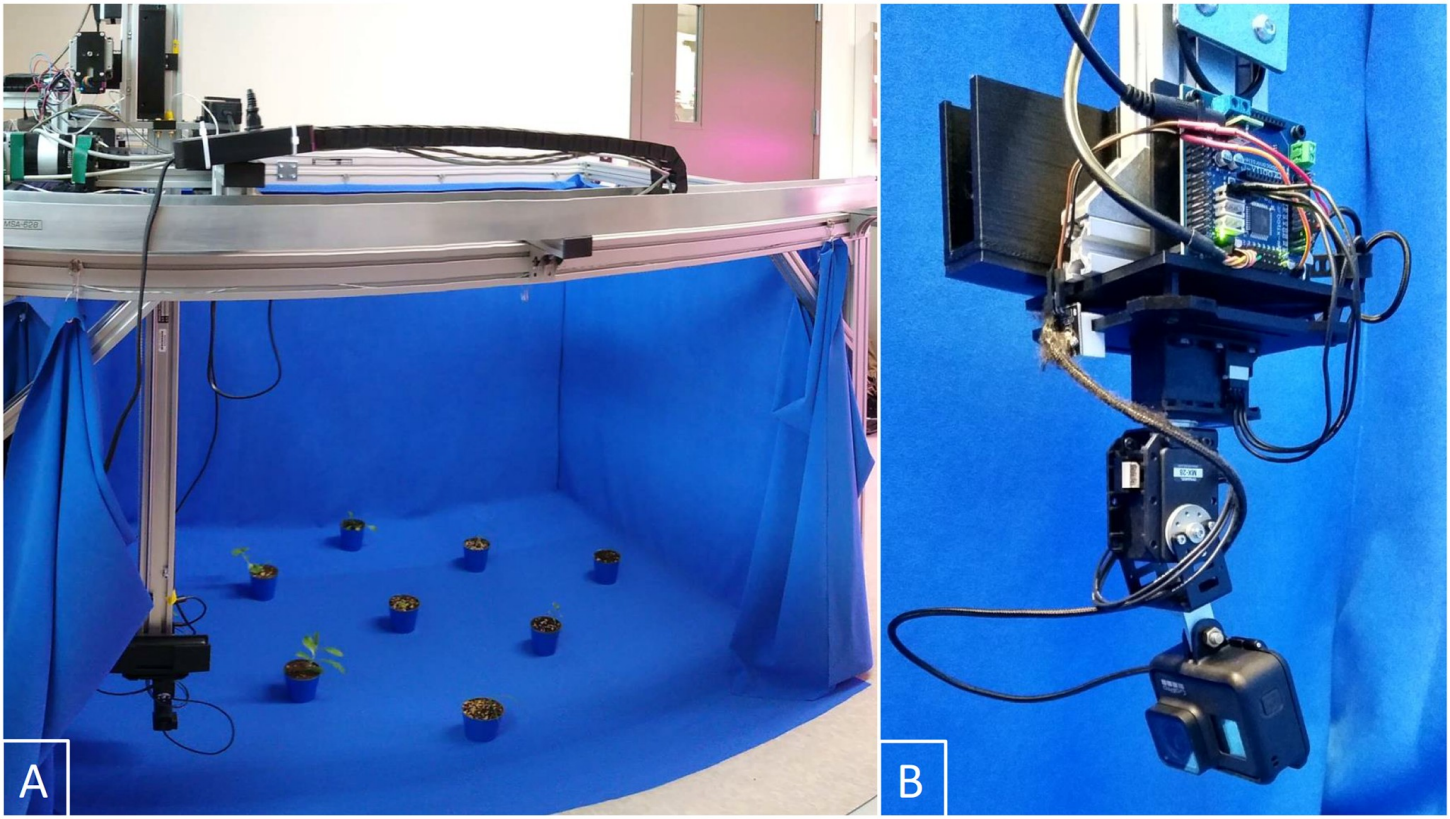
The EAGL-I system. A: Full view with blue keying fabric pulled back to show the imaging volume. B: Close-up view of the gantry head carrying the pan-tilt system, the camera, and a powerbank.

Table 1 gives an overview on the EAGL-I hardware. The system is setup in a gantry configuration (Fig 2), such that the gantry head can be moved in all three dimensions of a volume measuring 115 x 84 x 71 cm^3^. Two actuators per axis provide movement in the x-y-plane and a fifth actuator raises or lowers the gantry head. For safety and repeatability, we equipped the actuators with limit and homing switches. The normally closed limit switches prevent the actuators to move beyond their bounds. When the switches trigger (or lose power) the whole systems shuts off immediately and until a manual reset. The homing switches counteract possible drifts or slips of the actuators. An Arduino Uno controls the gantry system’s actuators, with power supplied by a 350-W AC/DC converter.

**Table 1:** System overview.

Part	Brand	Model	Specs	Notes
x, y, and z Actuators	Macron Dynamics	MSA-628	Travels: 1500 mm. (x), 1000 mm. (y and z)	Head moves in a volume of: 1150 × 840 × 718 mm^3^
Planetary Gearbox	ServoElements	MPS-60-005	5:1 Ratio	
Stepper Motors (xyz)	ServoElements	ST24-1.8-297	NEMA 24, 24 V DC 2.8 A 1.8° step angle 2.7 Nm rated torque	Motors have integrated stepper drives.
Controller of x, y, z Actuators	Arduino	Uno Rev3	Microcontroller: ATmega328PClock Speed: 16 MHz	Max. Pulse Rate: 4000 pulses/second
RGB-Camera	GoPro	Hero 7 Black	WiFi/Bluetooth controlled Res: 4000 × 3000 px FOV diagonal: 60.5°–149.2°	Used in linear mode, no zoom: FOV = 98.7° File format: jpg
Servo Motors (pan-tilt)	Dynamixel	MX-28T	11.1-14.8 V, 1.4 A 0.088° step angle 2.5 Nm stall torque	
AC/DC Converter	Mean Well USA	LRS-350-36	Output: 36 V, 9.7 A, 350 W	

On the gantry head we attached a pan-tilt system followed by an RGB camera. An Arduino-compatible micro-controller powers and controls the pan-tilt system via two servo motors, allowing the camera to be rotated through any combination of azimuthal and polar angles (360° pan, 180° tilt). The camera itself is powered by a commercial 20-Ah power bank that can support its imaging process for over 8 hours and is easily swapped out.

## 3 Data production

The two main contributions here are the duration of the robotic movement and the image processing time of the camera, each of which are discussed separately below.

### 3.1 Robotic movement

The camera is moved by the xyz-gantry and the pan-tilt-subsystem. Since panning and tilting the camera happens in parallel to the movement in x, y, and z (and is almost always faster), we can neglect that contribution for the imaging rate. We control the actuators close to the maximal pulse-rate the Arduino Uno can output (4000 pulses per second). This translates into a movement speed of

$$\begin{array}{c} v=p_{r}\cdot d\cdot s\cdot r\cdot m=p_{r}\cdot 0.105\cdot m, \end{array}$$ (1)

where *p*_*r*_ is the pulse rate, *d* = 105 is the distance traveled per revolution of the actuator in millimeters, *s* = 1.8/360 = 0.005 is the fraction of a full revolution made by 1 step of the stepper motors, *r* = 0.2 is the gearbox’s reduction ratio, and *m* is a factor determined by the stepping mode. For full-stepping mode *m* = 1, whereas half-stepping means *m* = 0.5. The controller uses a linear acceleration and deceleration profile to ease in and out of the actuators’ movements. Overall, then, we have a nearly linear proportional relationship between pulse rate and travel speed. Furthermore, all three axis can be moved in parallel or one after each other.

When the camera is moved to a new position and orientation, it is useful to pause before proceeding to trigger it to take an image. This allows vibrations to settle down and not doing so might result in blurry images, especially when using longer exposure times.

**Fig 3:**
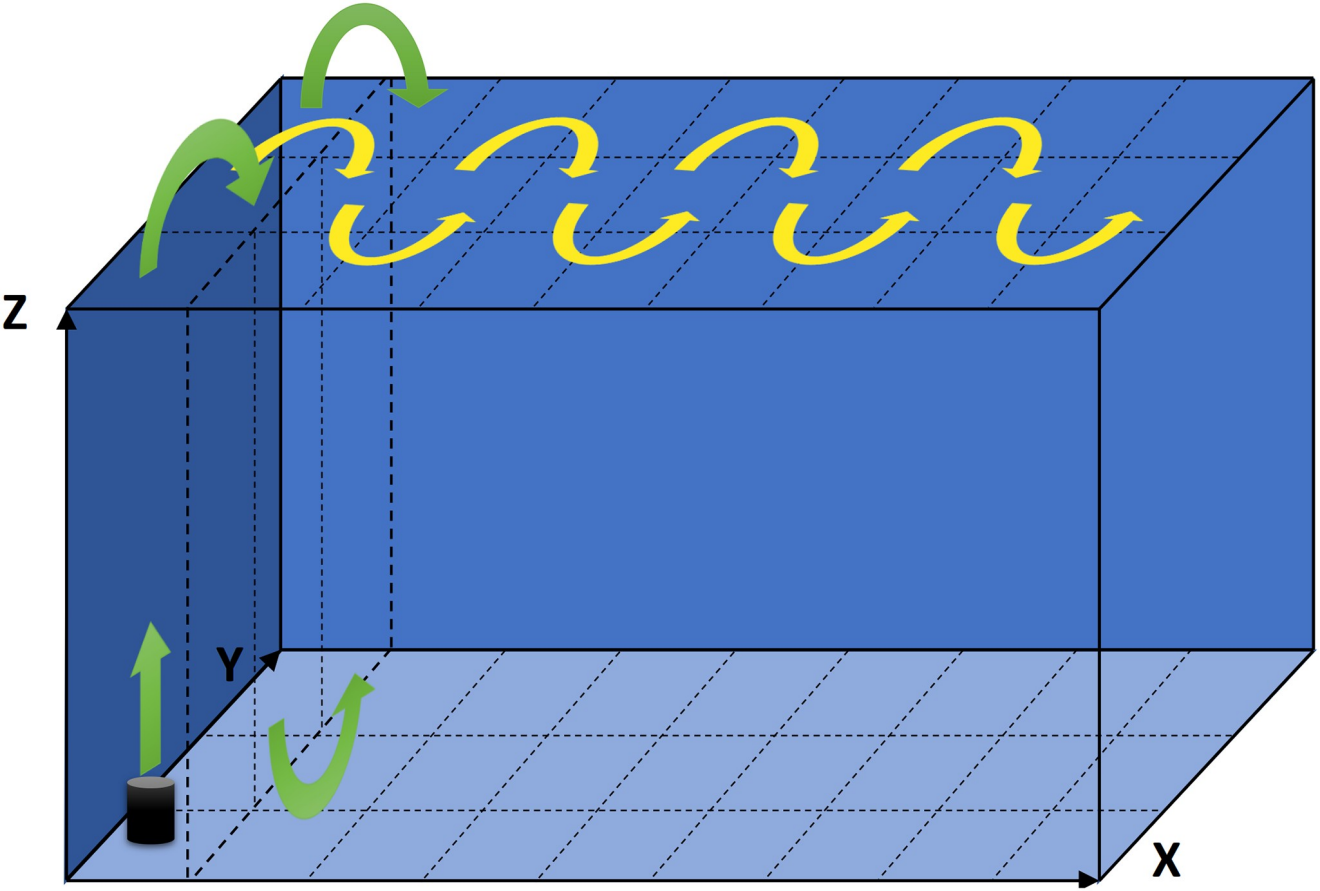
Path of gantry head. Movement of the gantry head in a zig-zag motion through columns and slabs of the coverable volume, starting in the bottom left near corner. The yellow arrows depict the motion from one slab to the next, nested inside those movements are the motions from one column to the next, depicted by the green arrows (only shown for the first slab).

When going through many different camera positions in sequence, the order in which those positions are visited is of equal, if not even higher, importance than the speed with which the camera is moved. To obtain a general optimal solution one would have to solve a three-dimensional traveling salesman problem (TSP), which is a well-known NP-hard problem in combinatorial optimization. In our typical application, we would have to solve the TSP for thousands of different positions. While still feasible, we settled for a nested zig-zag algorithm, as depicted in Fig 3, which offers a straightforward method to keep travel times between successive camera positions short.

The cuboid-shaped volume through which the gantry system can move the camera is divided into slabs of equal width along its x-axis. Those slabs are all subdivided into equally wide columns along the y-axis. Now, starting at the bottom of the first column (containing the coordinate system’s origin), we move the gantry head to the position inside that column with the smallest z-value. From there we move upwards through the positions with the next-largest z-values inside that column (ties in z-values are resolved arbitrarily). Note that small movements in x- and y-direction are still happening, but are limited by the columns boundaries. We keep moving upwards until reaching the highest position inside the column. From there we continue to the next column in positive y-direction and reverse the procedure: we start with the position having the largest z-value and descend through the column. We keep zig-zagging through the first slab’s columns until we reach the end of its last column. From there we move to the second slab in positive x-direction. We continue a zig-zag motion working our way through the columns, but this time, when we change columns we move in negative y-direction, until having traversed the entire second slab. We continue those zig-zag motions from slab to slab, until each position was visited.

### 3.2 Imaging process

The imaging process is initiated by sending an HTTP request to the camera over WiFi. The delay to send and process the request is negligible (of order of a few milliseconds) and thus is of no concern for the imaging rate. The time to perform the imaging itself depends on the camera settings and lighting conditions. In our indoor setup, without additional light sources and a maximal ISO of 200, the camera needs approximately 2.7 seconds to take an image. Allowing a higher exposure index would reduce that time, but also introduce grain to the image. Additional lighting will reduce the exposure time, but is presently not a main concern.

Images can be downloaded from the camera via a USB or WiFi connection. In either case, one can retrieve each image directly after it has been captured or retrieve all images in bulk after the system went through each of its positions. Retrieving the images in bulk decouples the imaging procedure from retrieving the data. By doing so, any delays or problems when transferring the images does not interfere with collecting the images. For the sake of automation, we value image collection higher than the data retrieval, since data generation takes much longer than its retrieval and thus is harder to repeat.

**Fig 4:**
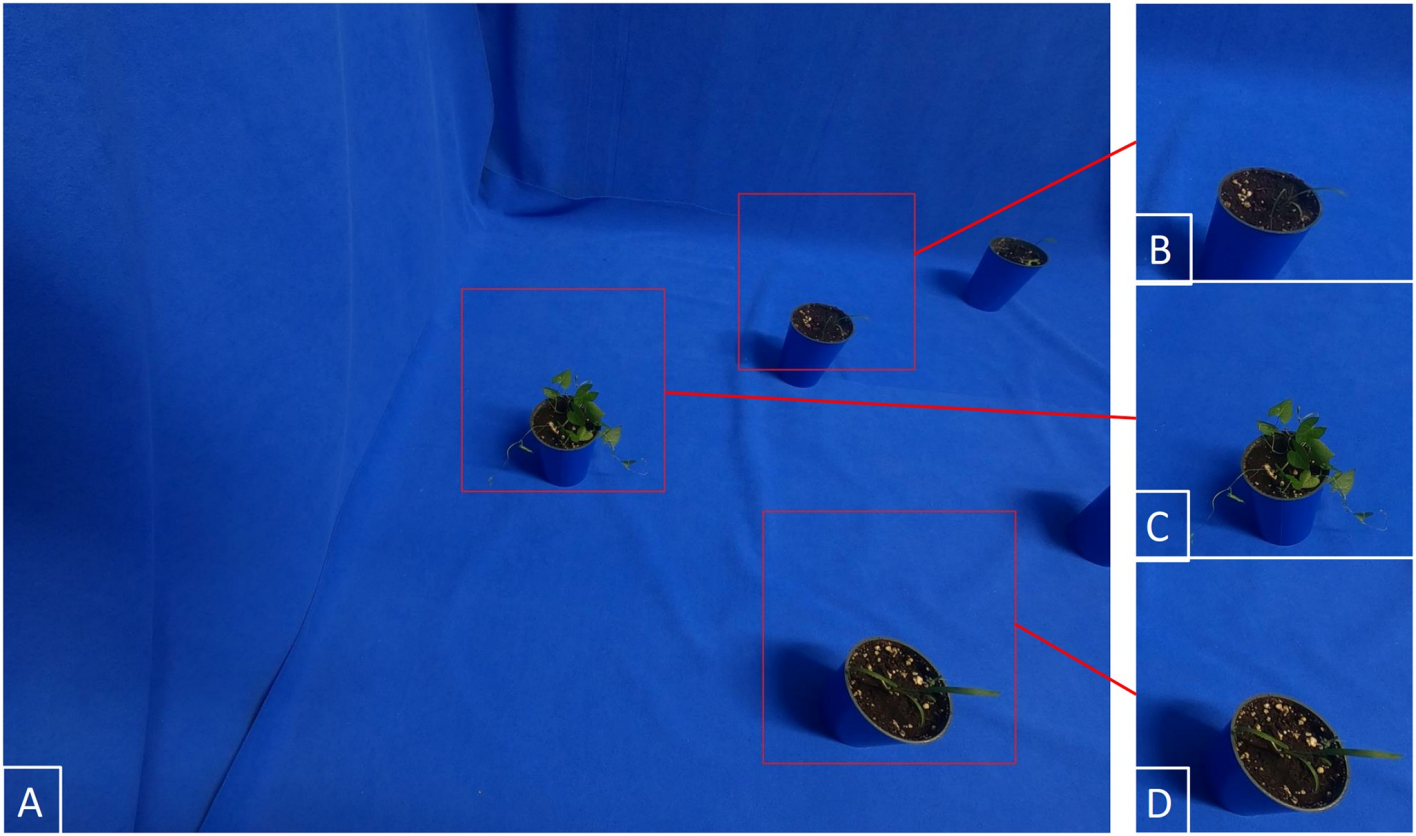
Master image and cropped images. A: Original master image taken by EAGL-I. B– D: three subimages cropped out from it. Note that the cropped images have different dimensions, whereas we present them here at the same size.

Depending on the application, an easy way to increase imaging rate is by cropping several subimages from a single image taken at a given position. In our application (generating single plant training data) this is a valid approach and can increase imaging rate up to one order of magnitude. Cropping out subimages results in different image sizes, which could be considered a drawback for some applications, but is rarely so in machine learning. Fig 4 shows an example of cropping several images from a master image.

### 3.3 Production settings

We define average production times *t*_*m*_ and *t*_*s*_ for master- and subimages, respectively, as follows:

$$\begin{array}{c} t_{m}=\frac{t_{p}+t_{d}}{N_{m}} \end{array}$$ (2)

$$\begin{array}{c} t_{s}=\frac{t_{p}+t_{d}+t_{c}}{N_{s}}\;, \end{array}$$ (3)

where *t*_*p*_ is the total time required to produce *N*_*m*_ master images (including robotic movements), *t*_*d*_ is the time to bulk download all master images from the camera to the computer, and *t*_*c*_ is the time required to crop out a total of *N*_*s*_ subimages from the master images.

To create a training dataset, we have performed runs with the system on a daily basis under the settings listed in Table 2. This resulted in *t*_*m*_ ∼ 7 s and *t*_*s*_ ∼ 4.8 s. Those settings are conservative and we have achieved during testing *t*_*s*_ < 1 s. Imaging at such fast rates comes at a cost of image quality, however. First, the shorter exposure time increases the ISO needed, which in turn introduces grain to the image. Second, to achieve maximal imaging rates, we have to pack plants in a tighter arrangement under the system. That can lead to overlap in the bounding boxes, i.e. meaning there are cases in which we can see plant material of neighboring plants in the images. Both points have to be accounted for, when using the data as training sets in machine learning. Higher grain in the image masks detailed features, and plant material from neighboring plants bring in unwanted features that do not correlate with the actual plant in the image. Image quality and imaging speed are two defining factors for the datasets that can be produced by EAGL-I and often have to be traded off for one another.

**Table 2:** Production settings.

Setting	Value
Locations Imaged	9
Parallel x, y, z Movement	No
Peak Pulse Rate	3000 pulses⋅s^−1^
Acceleration Rate	10000 pulses⋅s^−2^
Stepping Mode	Half-steps
Pause before Camera Trigger	3 seconds
Routing Algorithm	Nested Zig-Zag
Maximal ISO	200
Imaging Time	Approx. 2.7 seconds
Additional Lighting	None
Image Download	WiFi, In bulk
Total Images	2149
Total Subimages	3494
Time for Imaging *t*_*p*_	3 hours, 25 minutes
Download Time *t*_*d*_	46 minutes
Cropping Time *t*_*c*_	34 minutes
Imaging Rate (Images) *I*_*r*_	Approx. 7 s/image
Imaging Rate (Subimages) *I*_*c*_	Approx. 4.8 s/image
Size on Disk	8-9 GB

### 3.4 Cropping and labeling subimages

Different methods are available to us for cropping out a single plant from a master image. In the following we give a roadmap for two approaches based on image processing and CNNs, respectively. We chose for our system a third approach, instead, that relies on spatial information alone.

An image processing approach relies on color differences between the plant, the soil, and the image’s background. With segmentation algorithms we could identify the plants inside the image and construct a minimal bounding box around it. We describe a similar process in Subsection 2.5. In a second step the segments would have to be matched to the plants’ known positions to assign the correct label.

Alternatively one could consider machine learning techniques themselves for cropping and labeling subimages. This approach, however, can only be applied once a sufficiently trained model is available. Here a two-step procedure could be employed: First, a model is trained to define bounding boxes in the image for each plant. These bounding boxes would again be matched to the plants’ known positions for labeling. Now, a second model could be bootstrapped, that not only finds bounding boxes, but also labels them by recognizing the plants shown. Keeping in mind, that creating such models is ultimately the purpose of EAGL-I, we encounter a “chicken or egg” problem.

In the case that there are more than one plant captured in one image, both approaches mentioned above have to rely on the plants’ spatial information at one point or another to correctly match labels with subimages. Only after achieving the goal, which EAGL-I was built to solve, we can discard spatial information completely, while still correctly labeling subimages. On the other side, spatial information is always available to us and is *sufficient* for cropping and labeling subimages. This motivates the purely geometric approach we have implemented into our system. It calculates the plants’ coordinates inside the image from their known relative position and angle to the camera. As a result, labeling sub-images becomes trivial. Furthermore, the method is robust, as we do not have to rely on the stability of an image processing pipeline or a machine learning algorithm’s accuracy.

To calculate the bounding box around the plant we define a sequence of linear transformations that match the plant’s real-world coordinates (world frame) with the plant’s xy-position inside the image (image frame). The net transform is

$$\begin{array}{c} T=T_{w2c}\cdot T_{c2i}\;. \end{array}$$ (4)

Here *T*_*w*2*c*_ is the linear transformation from world frame to camera frame, i.e. a frame in which the camera is the origin pointing in positive x-direction. Thus, the linear transformation *T*_*w*2*c*_ consists of a translation, depending on the gantry head position and the displacement due to the pan-tilt system, and a rotation due to panning and tilting the camera. The transformation *T*_*c*2*i*_ converts the camera frame to the image frame, meaning that the objects inside the camera’s field of view are being projected on the xy-coordinates of the image. For this we calculate bearing and elevation of the object’s position from the camera. Using these angles we map the object to xy-coordinates (given in pixels), depending on the camera’s resolution and field of view. To calculate the object’s size in the image frame we calculate its subtended angle from the camera. To this end, we replace, for calculations, the plant by a sphere with radius large enough that the plant is contained inside of it. For full details on these transformations, we refer to our code in Ref. [67].

Given that we place plants on the floor (meaning the z-coordinate is known), we can also invert the projection *T*_*c*2*i*_ and the transform *T*_*w*2*c*_ to map the position and size of objects in image frame back to world frame. This inversion effectively allows us to determine any (sufficiently flat) object’s x- and y-position from a single overhead image taken by the system itself.

We want to point out that following a geometric approach to locate the plants comes with its own challenges: It relies on precise and accurate movements of the camera and location of the targets. Accuracy and precision of our robot’s movements turned out to be sufficient for this approach. To achieve good positioning of the targets, we measured and marked 12 target locations that we use repeatedly. The system can also generate new target locations and mark them with a laser. This allows us to not be limited to a fixed set of positions. A second challenge to a geometric approach are lens distortions, i.e. deviations from a perfect rectilinear projection from camera frame to image frame. Such distortions usually appear on the image frame’s margins. We countermeasure those drawbacks by using relatively large spheres to approximate the plants imaged. Other countermeasures would be to measure the distortions and use software correction before cropping the subimages, or to simply not use subimages that lie too close to the image’s margins, or to use digital zoom that effectively reduces the field of view to an area with only negligible lens distortions.

### 3.5 Image postprocessing

As mentioned above, EAGL-I produces images against a neutral blue background. This is a deliberate choice as the background has a high contrast with the plant matter, especially when converting the image’s colorspace from RGB to the CIELAB color space. This in turn, enables and simplifies image processing techniques, foremost background replacement. Having a background with little to no artifacts, or even a completely removed background, allows our data to be application-agnostic and thus be of use for a wider audience. By replacing the background users can tailor the data generated by the EAGL-I system towards their own application by, for example, inserting images of their local soil or lab environment as background.

**Fig 5:**
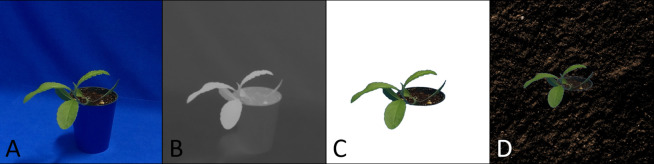
Background removal. A: Original Image captured by EAGL-I. B: The originals blue-yellow channel as a grayscale image. C: Keyed out image. The background is removed by defining a threshold for the blue-yellow values. All pixels below that threshold are masked out. D: The background is replaced by a stock photograph of soil.

We now shortly describe the background removal and replacement process for images created by our system (see Fig 5). We used the PlantCV library for Python [68], which itself is based on OpenCV [69]. In a first step we convert the image from the usual RGB color space to the CIELAB color space, in which the *b*-channel ranges from low values for blue pixels to high values for yellow pixels. Fig 5b shows the *b*-channel of our example as a grayscale image, the blue background appears dark, whereas the plant and soil are bright shades of gray. With a binary threshold-filter based on this channel we keyed out the plant as shown in Fig 5c. Additional filters can be applied to remove artifacts and to smooth the edges of the thresholding operation (e.g. dilating, filling holes, eroding, Gaussian blur). Once the background is keyed out, it can be replaced by any other application-specific background. In panel D of Fig 5 we used a stock photograph of soil as background. Again, additional measures can be taken to adjust for similar light conditions, perspectives and size. These are highly dependent on the application’s requirements.

Since the camera positioning can be repeated precisely, a second technique to key out the plant also becomes available: background subtraction. For this technique a second picture is taken from the same position and angle but without the plant. This image, that contains the background only, can be subtracted from the image containing the plant, leaving the plant itself.

Further image processing can be employed to remove the dark soil from the green plant or to extract morphological information. Those techniques are widely deployed in the area of (high throughput) phenotyping. For those techniques we refer to Ref. [68] and PlantCVs online documentation. For a visual demonstration of background removal and the EAGL-I system as a whole, we provide a supporting video, see S1 Video, with this paper.

## 4 The weedling dataset

As proof of concept we have generated a labeled dataset of seedlings of eight weeds that are common in Manitoban fields. This dataset [70] allows us to test systems that lie downstream in the development pipeline, in particular databases and the training of machine learning algorithms.

We chose weed species as targets, as they are of general interest and can be found amongst virtually every cash-crop in the field. The reasons to focus on a rather early growing stage are several. Using seedlings allows us to grow more individuals in rotation, discarding older plants for newer ones. This results in a richer dataset, compared to imaging a smaller number of individuals over their full life cycle. Furthermore, we can image more plants in parallel, thus achieving a higher imaging rate, if they are small. Lastly, a rather important and pragmatic argument is that the identification (and eradication) of weeds is most critical in the early stages of crop growth when plants are small and a canopy has not yet developed.

To generate the dataset we used the production settings as given in Table 2. In 10 runs we generated 34,666 subimages of weeds in a total imaging time of 47 hours and 30 minutes. Setting up the system to perform a single run requires personal attendance of roughly 15 minutes, after which the system continues autonomously and does not need further supervision. All images were taken in front of the blue background (Figs 1, 2 and 4) to ease image processing and segmentation. The dataset and its respective metadata (see below) is designed along two principles: First, we aim to showcase the system’s capabilities of taking images from many different perspectives, exceeding what would be possible by mere image processing. Second, along with its metadata this dataset should allow interested researchers to create subsets (e.g. only profile shots) that fit their needs and applications best. Table 3 gives an overview on the dataset’s characteristics.

**Table 3:** The weedling dataset.

Weed	Number of Images*
Echinochloa crus-galli (Barnyard Grass)	8621
Cirsium arvense (Canada Thistle)	4706
Brassica napus (Volunteer Canola)	6723
Taraxacum officinale (Dandelion)	4797
Persicaria spp. (Smartweed)	870
Fallopia convolvulus (Wild Buckwheat)	4621
Avena fatua (Wild Oat)	1218
Setaria pumila (Yellow Foxtail)	3110
Total	34,666

* Variations are due to different germination success

Each image of the dataset is accompanied by two additional files. The first is a copy of the original image that contains bounding boxes corresponding to the cropped out subimages. The second is a JSON-file containing the following metadata fields:

- *version*: A version number differentiating file formats; this dataset’s version is 1.5 and differs from earlier test sets in the number of data fields and formatting style.
- *file_name*: A unique image identifier of the form *yyyymmddhhmmss-pose#.jpg*, where the first 14 digits encode year, month, day, hour, minute, and second of when the image was captured. The number after *pose* denotes the position of a specific data-acquisition run.
- *bb_file_name*: A unique identifier for a copy of the master image with bounding boxes drawn on it. The format is equal to the one in *file_name* but with a *-bb* attached after the pose number.
- *date* and *time*: Date and time at which the picture was taken
- *room* and *institute*: Abbreviated location of where EAGL-I was set up.
- *camera* and *lens*: Information about the camera being used. In the case that there is no specific lens information the *lens* field can be used for model information (in our case we use *camera* = GoPro and *lens* = Hero 7 Black)
- *camera_pose*: A literal containing the camera position in terms of x, y, and z coordinates, polar-, and azimuthal angle.
- *bounding_boxes*: A list of objects containing information for all cropped subimages, containing the following fields for each such image:
  - *plant_id*: A unique identifier for each plant, consisting of the first letters of its scientific name and a number, for example: *echcru002*
  - *label*: The common name label, for example: *BarnyardGrass*
  - *scientific_name*: For example *Echinochloa crus-galli*
  - *position_id*: Denoting the positional ID at which the plant was located
  - *subimage_file_name*: A unique subimage identifier of the form *yyyymmddhhmmss#.jpg*, where # is the position ID that ensures uniqueness
  - *date_planted*: The day we put the plant’s seed in soil
  - *x_min*, *x_max*, *y_min*, *y_max*: The subimage’s coordinates in the parent image, given as a percentage. A value of *x* = 0, *y* = 0 denotes the image’s upper right corner, whereas *x* = 1, *y* = 1 denotes the lower left corner; this is conform to the directions as defined in the OpenCV-library, which is used for our image processing pipeline

**Fig 6:**
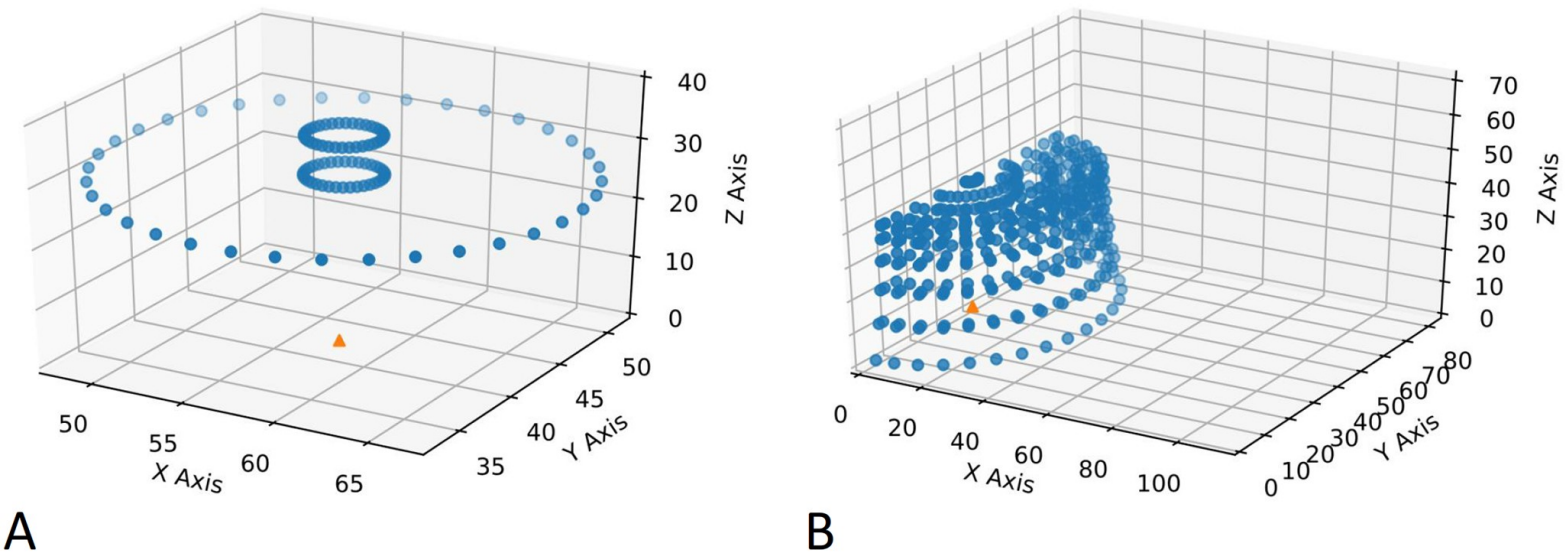
Camera positions. The different camera positions from which the plant located at the green triangle is imaged. Scenario A: Since the plant is located at the border of the traversable volume, we have a cylindrical shape from which we can image the plant. Scenario B: The plant is located in the inside of the traversable volume, resulting in circles at different heights and radii from which we image the plant.

Since the available imaging perspectives of a plant depends on where it is located, we have sorted the position IDs into two classes: In the first class, four of the positions lie on the edge of the volume that the gantry system can cover. That limits the camera-poses from which we can image that position to half a cylinder. The second class of positions lie in the interior of the coverable volume, resulting in a half-sphere of possible camera-poses to image from. See Fig 6 for a visualization of the two different classes. The subimages are sorted by these two location classes and saved into respective subfolders.

### 4.1 Application example: Training a simple CNN

To demonstrate the value of data collected with the EAGL-I system, we train a CNN that sorts plants into one of two distinct classes. We want to point out, that the task itself and the methods employed serve only as an example for how a dataset created by our system can be used. Achieving a model with an accuracy competitive to state-of-the-art deep learning methods lies outside the scope of this paper and will be the focus of future work. For more advanced models similar in structure we refer to Refs. [71–73]. Instead we take a data-centric perspective and employ the heuristic that supervised learning models are eventually limited by their training data [31, 39]. Consequently, the following discussion of results focuses on how EAGL-I can help in extending and modifying the training dataset to improve classification results for the model presented here.

#### 4.1.1 Model and task

The specific task we pose to the network is to differentiate between grasses and non-grasses. As representatives for grasses we have barnyard grass, wild oats and yellow foxtail. We chose this task (in contrast to other classification challenges like identifying each species by itself or for example differentiating the cash crop canola from weeds) for two reasons: First, a significant portion of our training images includes seedlings that have not grown their first true leaves, yet. Since all non-grasses in our datasets are dicots, a visual distinction between grasses and non-grasses is possible even during their earliest growing stages. Second, a key question to answer is how the data generation process has to be improved such that models trained on the respective data generalize to new scenarios. For this it is instrumental to test the models on external data. Defining this rather general task allows us to run the model with a wider variety of data, specifically to plants that we did not have access to when generating the training set.

To perform this task we trained a model based on the established ResNet architecture [74] with 50 layers and randomly initialized weights. We average and normalize the input images to enhance the actual differences between the pictures, which are the plants (in contrast to the rather uniform blue background). To counteract the slight imbalance between the two classes we introduce class weights *c*_*m*_ and *c*_*d*_ defined as

$$\begin{array}{c} c_{m}=\frac{\left|\text{Total}\;\text{images}\right|}{\left|\text{Images}\;\text{of}\;\text{monocots}\right|},\;c_{d}=\frac{\left|\text{Total}\;\text{images}\right|}{\left|\text{Images}\;\text{of}\;\text{dicots}\right|}. \end{array}$$ (5)

**Fig 7:**
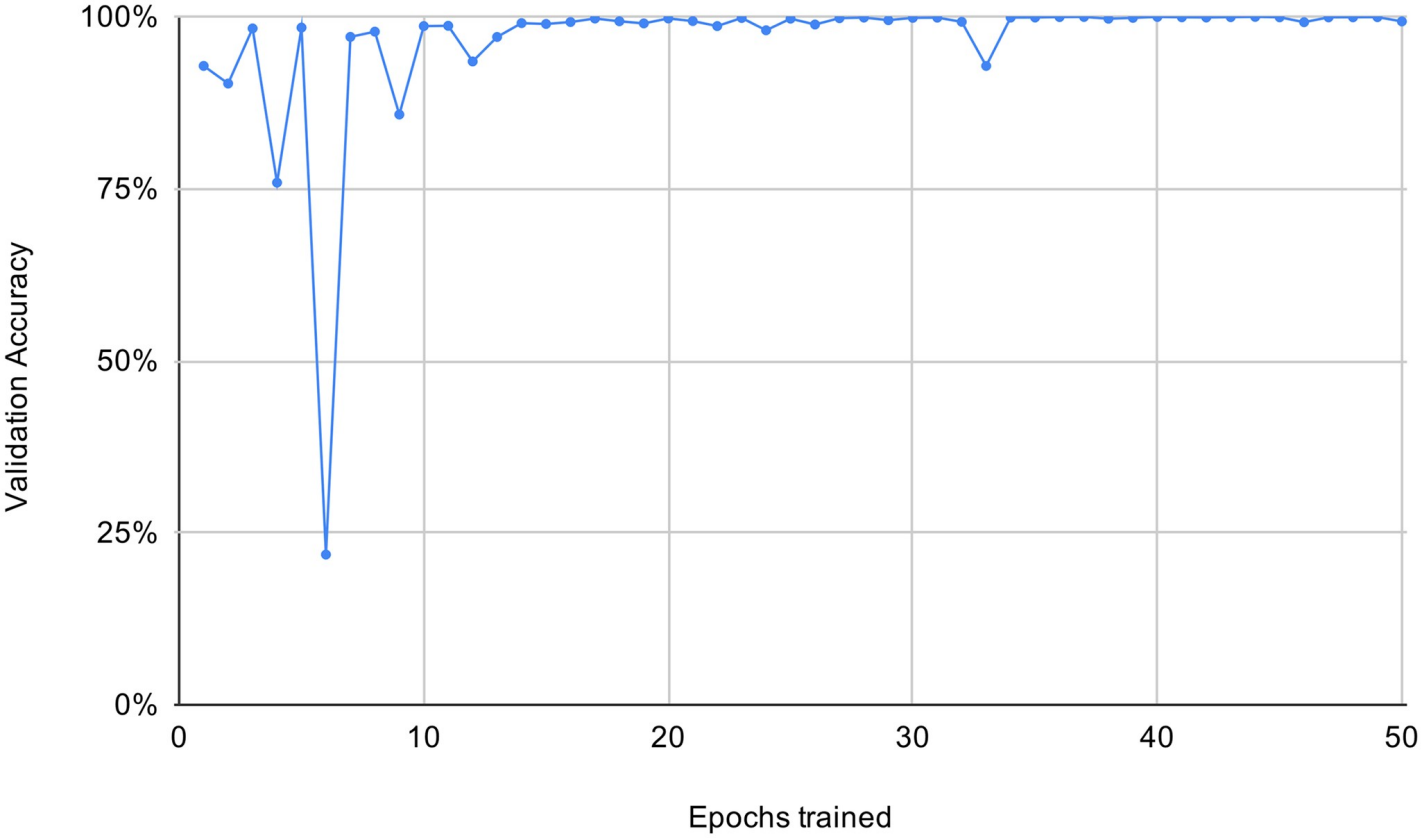
Validation accuracy in percent evaluated after each training epoch.

We used 80% of the data for training, reserved 20% as validation data, and repeated training over the entirety of the training set 50 times (each one forming an *epoch*). The validation accuracy achieved a satisfactory convergence with a validation accuracy of 99.71% after 50 epochs (average of 99.79% and a variance below 0.025% over the last 20 epochs). The evolution of the validation accuracy per trained epoch is graphed in Fig 7.

#### 4.1.2 Results in different scenarios

Now we present new data to our network. In doing so we test its capabilities and how well it generalizes to new scenarios. Furthermore, we discuss options on how the EAGL-I system can collect data that will lead to better models. As testing data we consider the following collections that increasingly differ from the training data:

- Images of the same species taken by the EAGL-I system, but with new individual plants. Those images differ from the training set only in having different individuals of the same species.
- Images of the same species taken by the EAGL-I system, but under randomized camera angles and distances.
- Images of the same species outside EAGL-I’s environment with a neutral background taken by a smartphone camera.
- A collection of Arabidopsis and tobacco plant images under lab conditions produced by Minvervi et al. [57].
- A collection of field data of sugar beets produced by Haug and Ostermann [75].
- A collection of plant seedling images produced by Giselsson et al. [76].

The results for the different scenarios are summarized in Table 4.

**Table 4:** Test datasets.

Test Dataset	Size of Test Set	Correctly Identified	Accuracy	Standard Error
Validation Dataset	6933	6913	99.71%	0.06%
EAGL-I camera, same species, same angles	3494	3437	98.4%	0.2%
EAGL-I camera, same species, randomized angles	520	513	98.7%	0.5%
Neutral Background, smartphone, same species	56	50	89.3%	4.1%
Minervi et al. [57]	347	283	81.6%	2.1%
Haug and Ostermann [75], field data, sugar beets	162 (of 494)	120	74.1%	3.4%
Giselsson et al. [76] field data, different species	500 (of 5539)	316	63.2%	2.2%

Before discussing the results, we point out that the test datasets are small compared to our training and validation datasets. This is a strong indicator that (i) the generation of good labeled datasets can indeed be time- and cost-intensive, and (ii) datasets are often associated with specific applications in mind. This reinforces why a system like EAGL-I is so important: It allows one to quickly produce large amounts labelled data for a wide variety of applications.

In the first scenario an accuracy of 98.4% was achieved, indicating that the model generalizes to new plants of the same species imaged under the same circumstances. The model we used has converged on the training data and might even show first signs of overfitting. For example, if we apply the model that is available after 40 epochs of training, the accuracy on the test data increases by 0.5% to 98.9%. To counteract overfitting and improving classification accuracy we can introduce more representatives of the two different classes to our dataset.

When we randomize the positions from which we take images, we see that it has no significant impact on the model’s overall accuracy. From this we conclude that the variety of angles covered in our training sets are sufficient for the model to be insensitive to imaging angles (such as profile shots or overhead shots) when distinguishing grasses from non-grasses.

For images taken by smartphone with a neutral background, a high accuracy above 89% is still achieved. The model generalizes to new imaging conditions, then, although with reduced accuracy (which is to be expected). Thus, the accuracy on the test data could deviate from the model’s accuracy on a larger set of similar images. To give a more complete picture of where the model’s true accuracy lies, we calculated a Clopper-Pearson confidence interval of [0.78, 0.96] at a confidence level *α* = 0.05.

We now explore how a model trained on our dataset generalizes to data produced by others for species that are not represented in our training set. The dataset in Ref. [57] consists of 283 images of Arabidopsis plants and 62 tobacco plant images. The images are all taken top-down and show the plants at different growing stages. The dataset was taken with phenotyping applications in mind and contains images of dicots only. On the overall data we achieve an accuracy of 81.6%, which in this case coincides with how many plants were classified as dicots. This is a strong demonstration that models trained with our data can generalize to species not included in the training data. If we break the test data down via the two species, we see that the model has an even better performance on the Arabidopsis images (91.2%), while performing rather poorly on tobaccos (37.1%). This tells us that the training set we generated is missing dicots that are morphologically similar to tobacco plants, and that we need to include these to achieve a more robust model.

As a next step to test how far our binary classifier generalizes, we applied it to the dataset provided in Ref. [75]. T his dataset consists of field data taken in a sugar beet field and features crop and weed plants. Since the annotations do not specify the weeds, we only use images that show sugar beets (a dicot). The original data in Ref. [75] shows several plants per image. Thus, we used the metadata provided by the authors to crop out the sugar beet plants. Still, on many of those cropped images we see plants overlapping into the cropped section. This is in contrast to our training data, which has all plants well separated from each other. The test data also features natural background (dirt) in contrast to the rather homogenous backgrounds on images we trained and tested on before. On the aforementioned subset our model achieves an accuracy of 74%. While not perfect, this shows that the model has already some capacity to generalize to new lighting and background conditions and another species of plants the model has not trained on. A first step to increase the usability of the training data for this application would be to include sugar beets into the training set. Also, positioning the plants closer to each other inside the EAGL-I system, such that overlaps happen on the resulting images, will result in training data more suitable for this task. Furthermore, the background can be replaced by images of soil typical for the fields in the test dataset.

**Fig 8:**
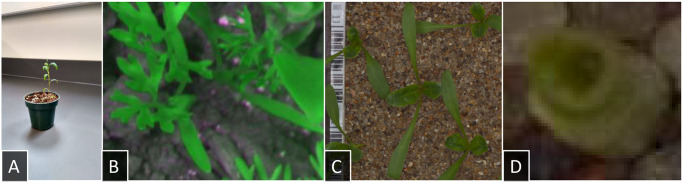
Examples of test data. A: A test image taken with a smartphone. B: A cropped out image from the dataset in Ref. [75]. C-D: Two examples from the seedling dataset of Ref. [76]: a high-res image containing multiple sugar beets and artifacts to the left border and a low-res image containing a maize seedling.

Finally, we applied our model to the dataset given in Ref. [76]. This dataset is very challenging for various reasons: First, the contrast between plant and background is not as distinguished as in our training set or the other test sets. Second, the data contains many plants at their earliest growing stages and as a result some images have a resolution as small as 49 x 49 pixels (see Fig 8 for an example of a high- and low-resolution image). Third, as in the previous test dataset, the images contain sometimes multiple overlapping plants, though the authors of Ref. [76] have ensured that only one species is present in each image. Fourth, the dataset contains only species that are not present in our training data. Still, our goal to distinguish monocots from dicots remains. To this end, we sorted the plants in Ref. [76] into two categories: maize, wheat, blackgrass, and loose silky-bent represent monocots; whereas sugar beet, mayweed, chickweed, shepherd’s purse, cleavers, charlock, fat hen, and cranesbill comprise the set of dicots. To test the model we chose the 250 images with highest resolutions for both classes. The achieved accuracy is 63.2% (confidence interval [0.59, 0.67] at *α* = 0.05). Although this value does not lie far above 50%, it is still significant as it shows that the model generalizes to some extent to data that shares only small similarities to the training data. A first step to improve accuracy would be to detect and crop out the plants in the test data before classification. This reduces the number of artifacts and ensures that no multiple plants are in a single image. Another improvement for this specific test data would be achieved by generating training data more suitable to the task, meaning imaging species used in Ref. [76] and focusing on overhead shots. As presented in Subsection 2.5, the blue background in the training images can be replaced by images of the granulate appearing in the images of Ref. [76] to achieve an even higher similarity to the test data. This idea to create training data that resembles the data we can expect in an application is exactly the raison d’etre of the EAGL-I system.

## 5 Conclusion and future work

In this paper we described the construction, operation, and utility of an embedded system (EAGL-I) that can automatically generate and label large datasets of plant images for machine learning applications in agriculture. Human interaction is reduced to selecting the plants to image and placing them inside the system’s imaging volume. EAGL-I can create a wide diversity of datasets as there are no limitations in plant placement, camera angle, or distance between camera and plant within this volume. Furthermore, the use of blue keying fabric as a background enables additional image processing techniques such as background replacement and image segmentation. The system’s performance was demonstrated along several dimensions. With a subimage production time of ∼4.8 s, we produced a dataset of over 34,000 labeled images of assorted weeds that are common in the Province of Manitoba. We subsequently used that dataset to train a simple convolutional neural network for distinguishing monocots from dicots, which in turn was tested on a variety of other datasets with quite favorable results.

We see the EAGL-I system as a important stepping stone to enabling new ML-based technologies in agriculture, such as automated weeding, that will require large amounts of labeled training data. Our system also provides opportunities to follow research questions that were not accessible before. For example, with the ability to generate a quasi-unlimited source of data ourselves, we can investigate how quantity and quality of training data influences machine learning models. Normally the amount of training data for a problem is hard-capped and acts as an observation limit for this type of research.

There are many other directions for improvements and future work for the EAGL-I system, of which we mention a few here.

### Future datasets

The EAGL-I system has been operational since late 2019. The Weedling dataset serves as a first sample and proof of concept for what the system can deliver. All the data that we eventually generate will be curated and released under a data management plan (see also Section 5). This includes partitioning the data into subsets similar to the Weedling dataset presented in Section 3.

### Lighting

The addition of programmable LED lighting elements are being planned and will allow us to customize lighting conditions on a per image basis, if desired. This will enable an even wider variety of images to be collected by simulating different lighting scenarios, e.g. sunny, cloudy, evening hours, etc.

### System design and dimensions

EAGL-I is presently limited to take images inside its coverable volume putting hard limits on the number and size of plants that can be imaged in a given run. This leads to research questions about the design of imaging systems that are specific for the creation of labeled data. The challenge, then, is to design a system that can produce a wide variety of images—preferably including a wide variety of plants differing in size and growing pattern—at a small cost and high imaging rate. The gantry architecture of EAGL-I is simple and functional, but may not be optimal. One direction we are considering is mounting linear actuators and cameras directly to the walls and ceiling of a growth chamber.

### Three dimensional plant data

Since we have full control over the camera position, we should be able to use software to reconstruct 3-dimensional plant models from 2-dimensional images taken from different angles. This could be a simple depth map extracted from two or more images via parallax or a 3d-point cloud combining more images. Alternatively, we can mount different imaging systems, such as stereoscopic cameras, to the gantry head in order to generate 3d data directly.

### Detection and imaging of plant organs

Often one is interested in the specific parts or organs of a plant, such as wheat spikes. To image these effectively, we have to solve how to point the camera at the desired organ for each plant. To achieve this we could combine machine learning techniques with our imaging system to bootstrap a training dataset for identifying specific plant organs. From there we can use a model to automatically move the camera in close proximity of the wheat spikes, say, and capture high resolution images. Both, the training set for identification, and the image dataset of high resolution wheat spikes would be valuable for subsequent applications such as phenotyping, blight detection and crop evaluation in the field.

### Scalability

We designed EAGL-I as our first concept to generate large quantities of labeled plant-image data. It is a simple gantry design that can be scaled up or down in size to meet the needs of the user. Multiple systems can also be employed to increase data generation rate. To this end, we are now developing more compact systems that trade imaging rate for lower costs and even easier operation. Such low-cost *plug-and-play* systems offer non-expert users the ability to generate bespoke datasets with minimal effort. The systems can be set up in standard growing chambers and research labs, allowing plant scientist and breeders to support their own work directly and at the same time contribute (if so desired) to communal data generation on an even larger scale than would be possible through just our present single EAGL-I system. Where possible, this additional data will be ingested into the overarching dataset mentioned above.

## 6 Data availability

The dataset and model described in Section 3 are publicly available [70]. The production of much larger future datasets is underway and will include Canadian crop plants, such as wheat, canola, soybean, and pulses. We presently envision depositing these datasets at the Federated Research Data Repository (https://www.frdr-dfdr.ca/repo/) through a data management plan developed with the tools provided by the Portage Network (https://portagenetwork.ca).

## Supporting information

S1 Video Video demonstrating EAGL-I and background removal. (MP4)
